# Supplementary material for: The Clinical Significance of CRNDE Gene Methylation, Polymorphisms, and CRNDEP Micropeptide Expression in Ovarian Tumors
Source: Int J Mol Sci. 2024 Jul 9;25(14):7531. doi: 10.3390/ijms25147531 (PMC11277161; doi:10.3390/ijms25147531)
Supplement: Supplementary file 1 [file ijms-25-07531-s001.zip › ijms-3010864-supplementary.pdf]

Supplementary Materials for

The Clinical Significance of *CRNDE* Gene Methylation, Polymorphisms, and CRNDEP Micropeptide Expression in Ovarian Tumors

Laura A. Szafron *et al.*

Corresponding authors' e-mails: laura.szafron@gmail.com, lukszafron@gmail.com

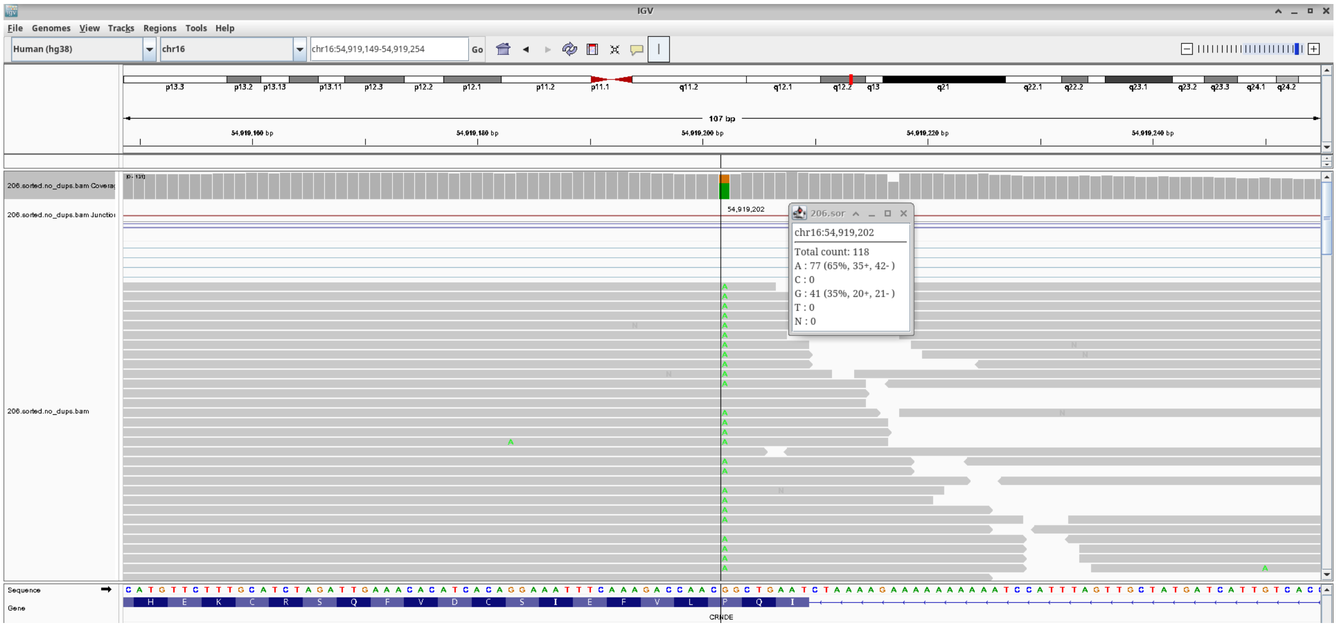

Fig. S1. An exemplary result showing the presence of a missense single nucleotide variant (SNV) in a hgOvCa tumor. This genetic alteration (GRCh38:chr16:g.54919202G>A, NP_001295892.1:p.Pro47Leu, rs115515594) leads to the change of the 47th amino acid residue in the CRNDEP micropeptide from proline to leucine. In the analyzed tumor, the given SNV was heterozygous, found in 77 reads, i.e., 65% of all the reads mapped to this locus. Remarkably, all optical and PCR duplicates are not included in this alignment.

Table S1. A multivariable Cox regression analysis showing that the rs115515594 SNP in *CRNDE* is a negative prognostic factor in BOTS, independent of genetic variants in the *PALB2* gene.

Formula	Data	Factor	HR	95% CI	p-value	N	Ev. no.	Best_time	AUC. MOM	AUC. UOM	AUC. MBM	AUC. UBM
Surv(RFS, Relapse == "Yes") ~ rs115515594 + Chemo-therapy + FIGO + Type + PALB2 + Age	full_table	**rs115515594: Present vs. absent**	**15.020**	[1.986-113.595]	**0.009**	53	6	1500	1.000	0.824	0.612	0.746
	full_table	FIGO: IC vs. IA-IB	> 100	[>100 - >100]	0.000	53	6	1500	1.000	0.824	0.612	0.746
	full_table	FIGO: IIA-IIIC vs. IA-IB	> 100	[>100 - >100]	0.000	53	6	1500	1.000	0.824	0.612	0.746
	full_table	Type: serous vs. other	0.098	[0.011-0.853]	0.035	53	6	1500	1.000	0.824	0.612	0.746

RFS – relapse-free survival, Type – histological type; HR – hazard ratio; CI – confidence interval; Ev. no. – events no.; AUC – area under a ROC curve; MOM – multivariable original model; UOM – univariable original model; MBM – multivariable bootstrapped model; UBM – univariable bootstrapped model; Best_time – follow-up time in days with the highest AUC value for the MBM.

Table S2. Genetic variants in linkage disequilibrium with the rs115515594 SNP in the European population of healthy individuals, assessed with the LDproxy app from the LDlink suite.

RS_Number	Alleles	MAF	Distance	Dprime	R2	Correlated _Alleles	FORGE db	Regulome DB	Functional Class
**rs115515594**	**(G/A)**	**0.004**	**0**	**1**	**1**	**G=G,A=A**	**5**	**7**	**Missense (Ensembl v. 100)***
rs1963030529	(-/A)	0.007	-92196	0.7482	0.319	G=-,A=A	NA	.	NA
rs568337223	(C/A)	0.001	-59673	1	0.2493	G=C,A=A	NA	.	NA
rs564180731	(G/A)	0.001	70930	1	0.2493	G=G,A=A	NA	.	NA
rs546830686	(A/G)	0.001	108994	1	0.2493	G=A,A=G	NA	.	NA
rs558978743	(A/G)	0.001	-133575	1	0.2493	G=A,A=G	1	.	NA
rs139807422	(A/G)	0.001	135558	1	0.2493	G=A,A=G	6	.	NA
rs546620788	(C/T)	0.001	-223293	1	0.2493	G=C,A=T	NA	.	NA
rs547053997	(G/A)	0.001	-224588	1	0.2493	G=G,A=A	NA	.	NA
rs79791443	(T/C)	0.001	-227603	1	0.2493	G=T,A=C	4	5	NA
rs142772261	(T/C)	0.001	-230960	1	0.2493	G=T,A=C	4	.	NA
rs139476806	(C/T)	0.001	-231194	1	0.2493	G=C,A=T	2	.	NA
rs148759424	(A/G)	0.001	-234289	1	0.2493	G=A,A=G	5	.	NA
rs577448266	(A/C)	0.001	272604	1	0.2493	G=A,A=C	NA	.	NA
rs189729447	(C/T)	0.001	-290138	1	0.2493	G=C,A=T	5	.	NA
rs527521719	(A/G)	0.001	297562	1	0.2493	G=A,A=G	NA	.	NA
rs547019981	(G/A)	0.001	361718	1	0.2493	G=G,A=A	1	.	NA
rs531083438	(G/A)	0.001	431343	1	0.2493	G=G,A=A	NA	.	NA
rs144177549	(T/C)	0.001	432556	1	0.2493	G=T,A=C	6	.	NA
rs540950886	(G/T)	0.001	-449905	1	0.2493	G=G,A=T	1	.	NA
rs527879717	(T/A)	0.001	-468987	1	0.2493	G=T,A=A	NA	.	NA
rs141564173	(C/T)	0.0089	-182153	0.7477	0.2473	G=C,A=T	5	5	NA
rs117426645	(C/G)	0.0169	-70670	1	0.2322	G=C,A=G	5	5	NA
rs117089870	(T/A)	0.0099	-107645	0.7475	0.2222	G=T,A=A	8	.	NA
rs116999486	(G/A)	0.0258	39164	1	0.1505	G=G,A=A	5	4	NA
rs141385587	(A/T)	0.007	382555	0.4965	0.1404	G=A,A=T	5	.	NA
rs566085121	(T/C)	0.007	414759	0.4965	0.1404	G=T,A=C	1	.	NA
rs146970803	(C/T)	0.007	421877	0.4965	0.1404	G=C,A=T	4	.	NA
rs150079469	(C/T)	0.007	467930	0.4965	0.1404	G=C,A=T	7	.	NA
rs117633800	(C/G)	0.0288	11092	1	0.1345	G=C,A=G	8	2b	NA
rs117450462	(G/A)	0.0298	20577	1	0.1299	G=G,A=A	8	4	NA
rs147190559	(A/G)	0.002	-240268	0.498	0.1238	G=A,A=G	4	.	NA
rs149269085	(C/T)	0.002	-263794	0.498	0.1238	G=C,A=T	8	.	NA
rs8053072	(A/G)	0.002	-266998	0.498	0.1238	G=A,A=G	6	4	NA
rs80019321	(C/A)	0.002	-268869	0.498	0.1238	G=C,A=A	5	3a	NA
rs541519938	(C/G)	0.002	274607	0.498	0.1238	G=C,A=G	NA	.	NA
rs149120690	(T/A)	0.002	294005	0.498	0.1238	G=T,A=A	4	.	NA
rs139300665	(T/C)	0.002	302527	0.498	0.1238	G=T,A=C	4	.	NA
rs562518413	(T/C)	0.002	308657	0.498	0.1238	G=T,A=C	NA	.	NA
rs150669506	(C/G)	0.008	449558	0.496	0.1225	G=C,A=G	4	.	NA
rs114533899	(G/T)	0.0089	384715	0.4955	0.1086	G=G,A=T	4	5	NA

A SNP with the highest probability of being a functional variant, i.e., playing regulatory roles in the cells, (rs117633800, determined by a high FORGEdb and a low RegulomeDB score, respectively) is highlighted in yellow. All variants in linkage disequilibrium are located in non-coding regions of the genome, whereas rs115515594 occurs in the coding region of *CRNDE*. * – in the 100th version of the Ensembl database, rs115515594 is classified as a missense variant, while in newer versions, the CRNDEP peptide in not included.

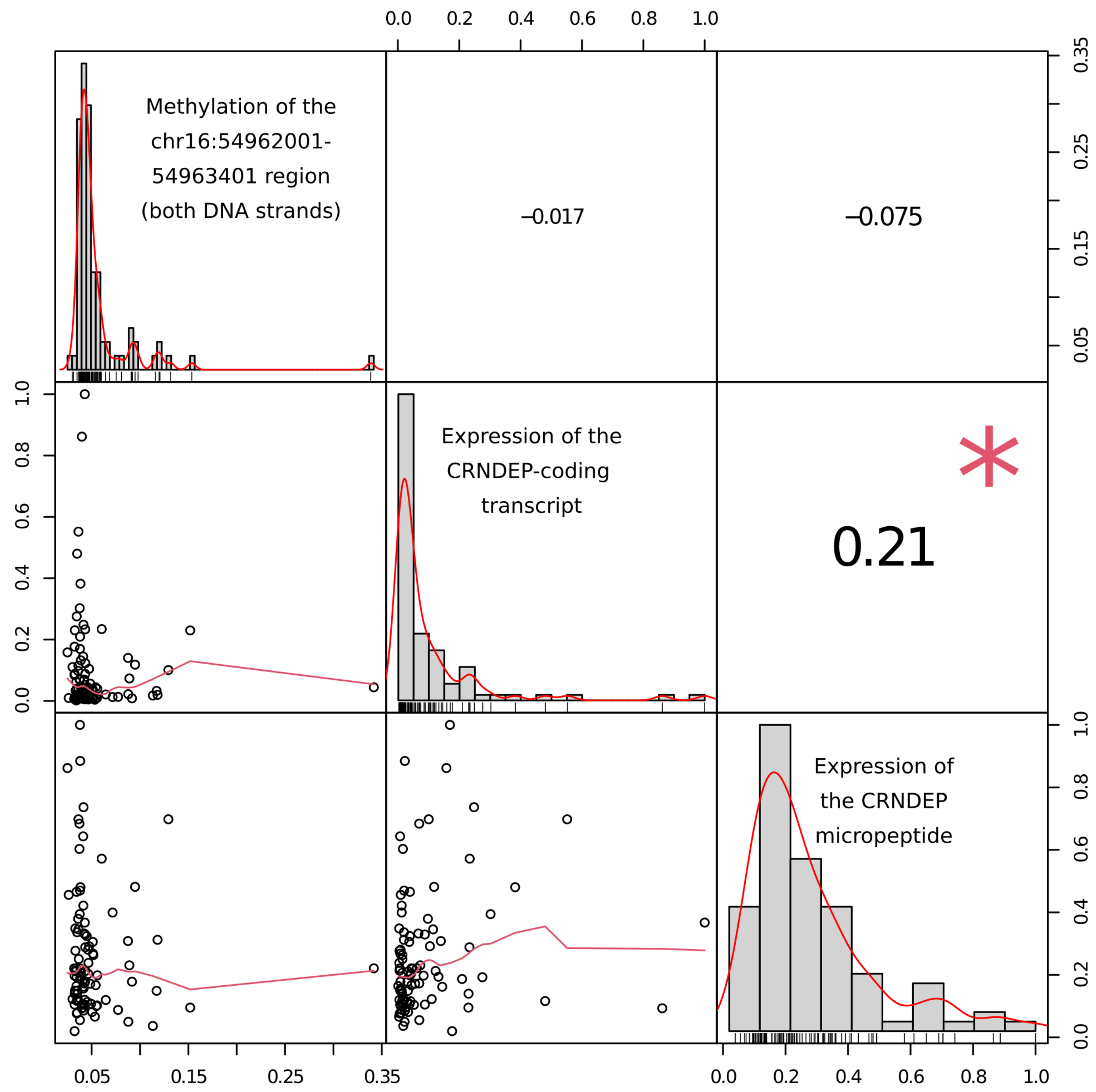

Fig. S2. The Spearman's correlation matrix for *CRNDE* gene methylation, expression and CRNDEP levels in 92 OvCa samples. The results are displayed at the intersections of tiles with variable names and histograms. On the bottom left side of the graph, the bivariate scatter plots with fitted lines (red) are shown, whereas the correlation coefficient (r) values are placed on the top right side of the graph. If the correlation for the given comparison is statistically significant ($p < 0.05$), the r value is accompanied by a red star.

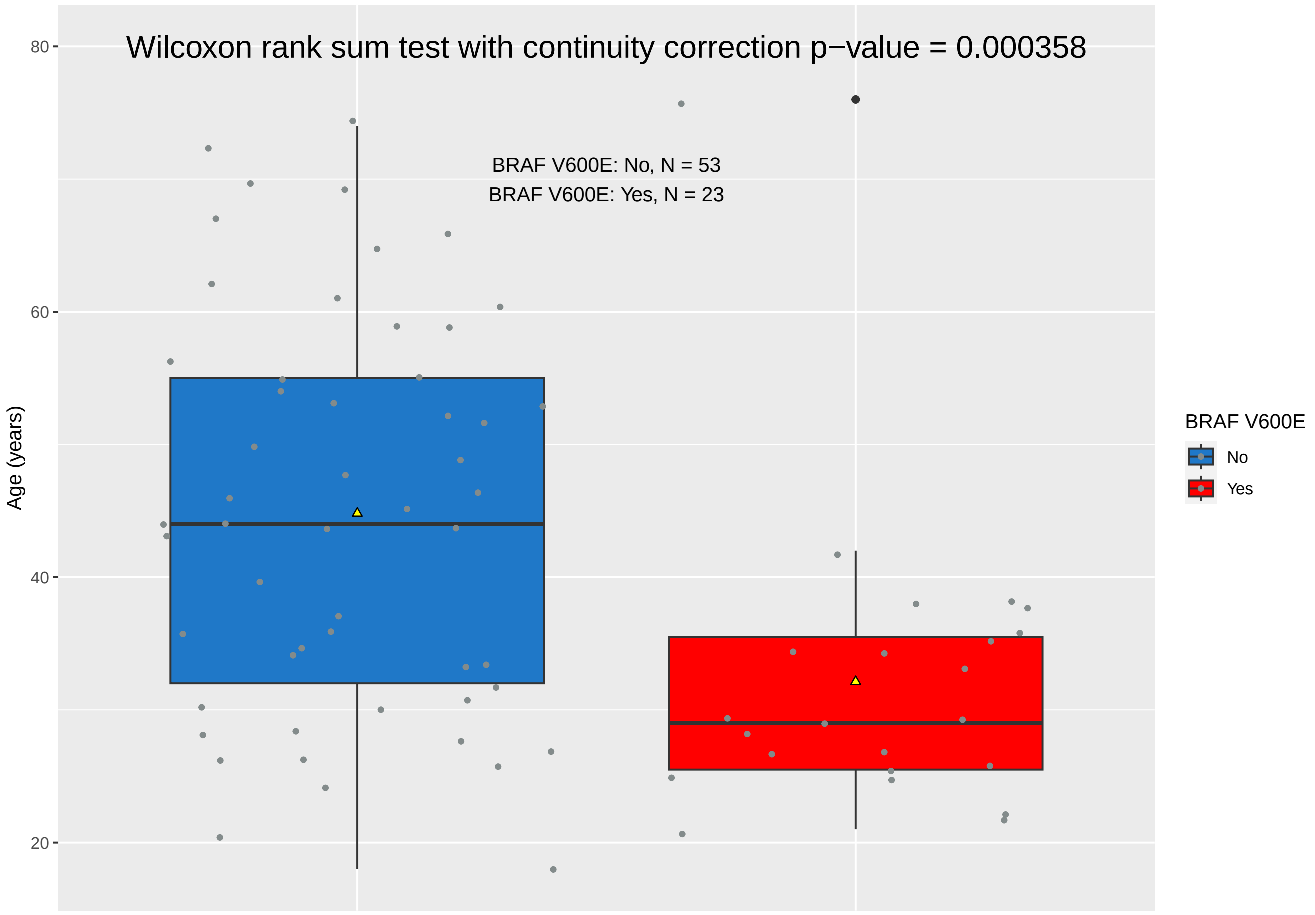

Fig. S3. Differences in the age at diagnosis in patients with borderline ovarian tumors with or without the *BRAF* V600E mutation. The boxplot is supplemented with mean values (yellow triangles) and the result of the Wilcoxon rank sum test.

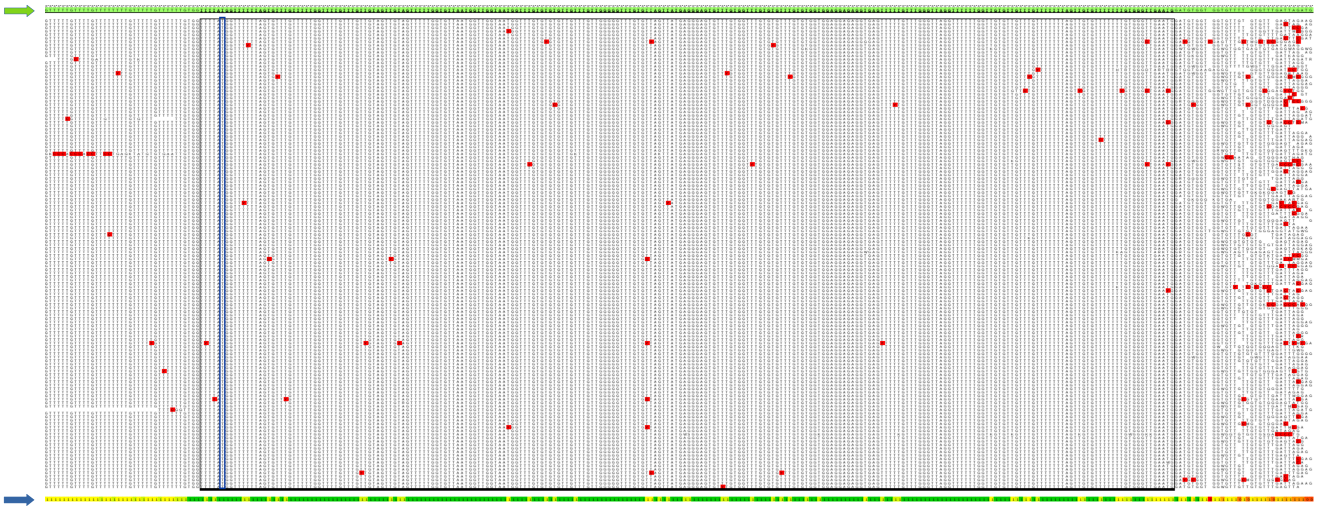

Fig. S4. The analysis of a large CpG island, GRCh37:chr16:54,962,540-54,962,906 (on a negative DNA strand), encompassing the promoter and the first exon of *CRNDE*. This evaluation was performed with methylation-specific PCR followed by Sanger sequencing. The analyzed region consisted of 367 bp and contained 63 CpG sites. This investigation, carried out on 134 OvCa samples, proved the existence of methylated cytosines within this region, that are not covered by microarrays used in our study. A single CpG site, the methylation status of which could be assessed with these microarrays, GRCh37:chr16:54,962,832, is marked with a blue rectangle. Red dots represent methylated cytosines. With a green arrow, a reference DNA sequence is marked. A blue arrow indicates the consensus sequence, where the green color denotes a perfect fit between all studied tumors and the reference sequence (no methylation of the given nucleotide), while the red color means no fit (all samples would have this nucleotide methylated). With a black frame, a region consisting of 232 nucleotides is marked. Within this region, good-quality chromatograms were obtained for all the samples, giving us the opportunity to perform a detailed bioinformatic analysis of all CpG sites present in this area.

Table S3. A list of antibodies used in the present study.

#	Detected protein	Immunized species	Manufacturer	Catalog no.	Concentration /amount used (method)	Antibody type/ conjugate
1	TP53	Mouse	Sigma-Genosys	PAb1801	1:500 (IHC)	I/-
	Mouse igG	Goat	Immunotech	816	1:1,500 (IHC)	II/biotin
2	CRNDEP	Rabbit	Abgent Inc.	custom-made	1:100 (DB); 1:800 (IHC)	I/-
3	Rabbit IgG	Goat	Thermo	31462	1:10,000 (DB)	II/HRP
4	Rabbit IgG	Goat	Beckman Coulter	IM0309	1:1,500 (IHC)	II/biotin

DB – dot blot; IHC – immunohistochemistry; HRP – horseradish peroxidase; I – primary antibody; II – secondary antibody.

Table S4. Clinico-pathological characteristics of the BOTS group.

	CRNDE var.			*CRNDE* DMR			CRNDEP exp.		
**BRAF.V600E**	**No**	**Yes**	**All samples**	**No**	**Yes**	**All samples**	**No**	**Yes**	**All samples**
**N**	52	22	74	27	20	47	16	5	21
**Microinvasion_or_Implants.No**	35	13	48	16	11	27	9	2	11
**Microinvasion_or_Implants.Yes**	17	9	26	11	9	20	7	3	10
**Relapse.No**	46	19	65	23	17	40	12	4	16
**Relapse.Yes**	6	3	9	4	3	7	4	1	5
**RFS.Min.**	112	293	112	178	293	178	112	293	112
**RFS.1st Qu.**	3668.8	2880.3	3271.3	3120.5	2805	2898	2517	2730	2730
**RFS.Median**	4677.5	4331.5	4539.5	4955	4331.5	4557	4340	2830	4245
**RFS.Mean**	4453.8	4033.7	4328.9	4400.9	4009.2	4234.2	3808.5	3169.6	3656.4
**RFS.3rd Qu.**	5488	5515	5523.3	5521.5	5576.3	5586.5	5071.3	4245	4955
**RFS.Max.**	7205	6190	7205	7085	6190	7085	6870	5750	6870
**Chemotherapy.No**	40	18	58	21	16	37	9	4	13
**Chemotherapy.Yes**	12	4	16	6	4	10	7	1	8
**Primary_tumor.No**	13	7	20	8	7	15	3	0	3
**Primary_tumor.Yes**	38	15	53	19	13	32	13	5	18
**Primary_tumor.NA**	1	0	1	0	0	0	0	0	0
**FIGO.IA-IB**	13	5	18	4	3	7	3	1	4
**FIGO.IC**	14	5	19	7	5	12	6	1	7
**FIGO.IIA-IIIC**	11	5	16	8	5	13	4	3	7
**FIGO.NA**	14	7	21	8	7	15	3	0	3
**Type.other**	14	0	14	NA	NA	NA	5	0	5
**Type.serous**	38	22	60	27	20	47	11	5	16
**Age.Min.**	18	21	18	18	21	18	26	29	26
**Age.1st Qu.**	31.8	26.3	28.3	28	25.8	27	45.5	35	36
**Age.Median**	44	29	36.5	44	29	34	51.5	36	46
**Age.Mean**	44.5	32.6	41.0	43.6	32.4	38.8	51.7	35.2	47.8
**Age.3rd Qu.**	55	35.8	52	54.5	35.3	46	62.8	38	60
**Age.Max.**	74	76	76	74	76	76	74	38	74
**Frozen_samples**	16	5	21	11	5	16	16	5	21
**FFPE_samples**	36	17	53	16	15	31	NA	NA	NA

RFS – relapse free survival; Type – histological type; Qu. – quartile; NA – not applicable/missing data; *CRNDE* var. – group of tumors in which genetic variants in *CRNDE* were evaluated; *CRNDE* DMR – group of tumors in which methylation changes in the differentially methylated region within *CRNDE* were assessed; CRNDEP exp. – group of tumors in which CRNDEP expression was measured.

Table S5. Clinico-pathological characteristics of the OvCa group.

	CRNDE DMR					*CRNDE* var./CRNDEP exp.				
**Therapy**	**PC**	**PC**	**TP**	**TP**	**All samples**	**PC**	**PC**	**TP**	**TP**	**All samples**
**TP53.accumulation**	No	Yes	No	Yes		No	Yes	No	Yes	
**N**	9	13	31	50	103	12	23	42	70	147
**CR.No**	4	5	10	13	32	4	9	15	16	44
**CR.Yes**	5	8	21	37	71	8	14	27	54	103
**Relapse.No**	0	1	2	5	8	1	1	4	8	14
**Relapse.Yes**	5	7	19	32	63	7	13	23	46	89
**Relapse.NA**	4	5	10	13	32	4	9	15	16	44
**Death.No**	0	1	2	9	12	0	1	4	14	19
**Death.Yes**	9	12	29	41	91	12	22	38	56	128
**PS.No**	4	9	13	17	43	4	14	19	21	58
**PS.Yes**	5	4	18	33	60	8	9	23	49	89
**DFS.Min.**	0	0	0	0	0	0	0	0	0	0
**DFS.1st Qu.**	0	0	0	22.5	0	0	0	0	139.8	0
**DFS.Median**	226	127	237	381	229	266	127	230.5	363	251
**DFS.Mean**	270.4	337.6	288.5	516.3	403.7	454.3	384.1	387.5	548.3	469.0
**DFS.3rd Qu.**	523	222	458.5	727	562.5	548	460.5	484.3	743.5	591.5
**DFS.Max.**	815	2521	1205	2452	2521	2426	2521	4380	2884	4380
**OS.Min.**	56	81	263	296	56	56	81	263	296	56
**OS.1st Qu.**	467	443	620	891.3	634.5	497.8	587.5	655.75	876.5	683
**OS.Median**	1138	687	853	1236	1066	1167	897	853.5	1185.5	1105
**OS.Mean**	1196.3	925.7	1168.4	1554.8	1327.8	1293.2	1186.1	1271.9	1571.2	1402.7
**OS.3rd Qu.**	1955	1318	1376.5	1953.3	1765.5	2046.3	1578	1706.3	1982.3	1864
**OS.Max.**	2742	2801	3343	5630	5630	2742	3750	4500	5630	5630
**Type.other**	NA	NA	NA	NA	NA	0	1	11	15	27
**Type.serous**	9	13	31	50	103	12	22	31	55	120
**FIGO.IC-IIC**	0	0	1	1	2	0	0	2	2	4
**FIGO.IIIA-IIIB**	1	2	4	4	11	1	6	4	5	16
**FIGO.IIIC**	5	10	24	42	81	8	15	34	58	115
**FIGO.IV**	3	1	2	3	9	3	2	2	5	12
**Grade.lgOvCa**	4	0	3	0	7	4	0	4	0	8
**Grade.hgOvCa**	5	13	28	50	96	8	23	38	70	139
**RT = 0 cm**	2	1	6	10	19	3	5	9	18	35
**RT < 2 cm**	4	3	20	28	55	4	6	26	35	71
**RT ≥ 2 cm**	3	9	5	11	28	5	12	7	16	40
**RT.NA**	0	0	0	1	1	0	0	0	1	1
**Frozen_samples**	8	13	28	48	97	12	23	42	70	147
**FFPE_samples**	1	0	3	2	6	NA	NA	NA	NA	NA
**Age.Min.**	34	42	29	33	29	34	36	20	33	20
**Age.1st Qu.**	43	55	49.5	47	47	44.5	45.5	47.5	47	47
**Age.Median**	48	60	54	54	55	49	58	53	53	53
**Age.Mean**	52.6	58.4	53.5	54.5	54.5	53	55.7	52.6	54.3	53.9
**Age.3rd Qu.**	64	65	62	62.5	63.5	64.3	64	61.5	61	62.5
**Age.Max.**	68	77	74	84	84	68	77	74	84	84

CR – complete remission; PS – platinum sensitivity; DFS – disease-free survival; OS – overall survival; Type – histological type; RT – residual tumor size; PC – platinum/cyclophosphamide; TP – taxane/platinum; NA – not applicable/missing data; Qu. – quartile; *CRNDE* DMR – group of tumors in which methylation changes in the differentially methylated region within *CRNDE* were assessed; *CRNDE* var. – group of tumors in which genetic variants in *CRNDE* were evaluated; CRNDEP exp. – group of tumors in which CRNDEP expression was measured.

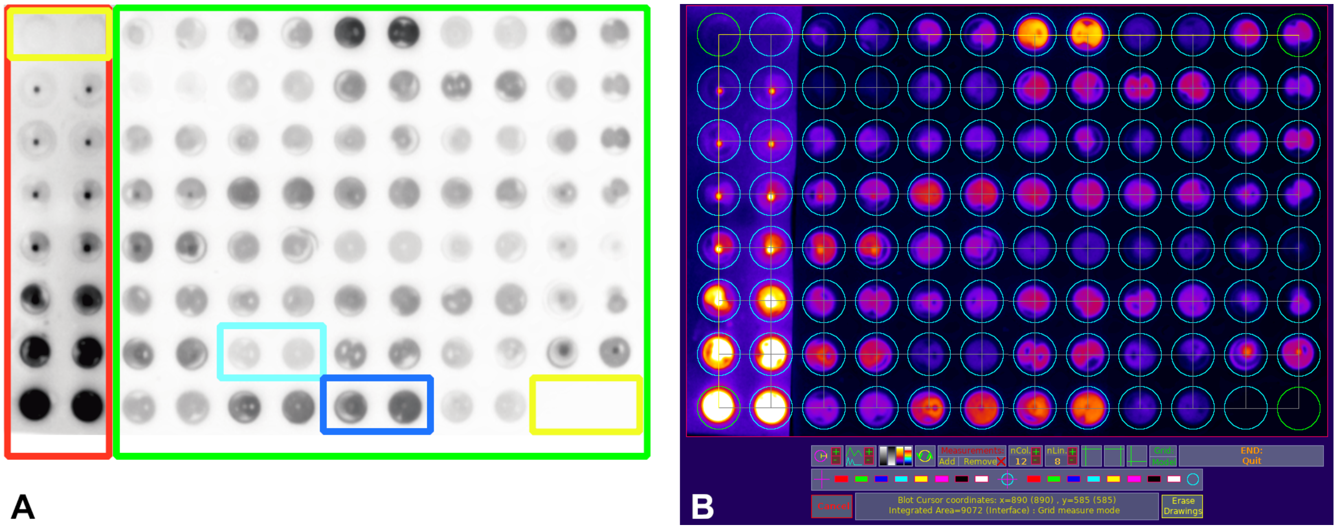

Fig. S5. An exemplary dot blot result of the CRNDEP expression analysis. In Fig. A, the original image of a nitrocellulose membrane is displayed. A red rectangle confines the region were known, gradually decreasing concentrations of bovine serum albumin (BSA) were added to obtain a standard curve, subsequently used in the expression analysis. In the area encompassed by the green rectangle, the expression of CRNDEP was assessed. Two different OvCa samples characterized by either moderate (light blue) or strong (dark blue) CRNDEP expression were utilized as calibrators. In both BSA- and CRNDEP-related analyses, negative controls (blank samples) are marked with yellow rectangles. Every sample was analyzed in two horizontally-oriented replicates. In Fig. B, a heatplot corresponding to Fig. A is shown. This plot, generated in the Protein Array Analyzer plugin for imageJ, depicts circular regions in which chemiluminescence signal was analyzed for every well.

Table S6. A list of PCR primers utilized in the present study.

Molecule name	Sequence (5' → 3')	Description
CRNDEmF4	GTTTTTTATTAGTTTAAGGATTTGAGTTAT	Primers used in methylation-specific PCR and Sanger sequencing reaction (CRNDEmF4) to verify the methylation pattern of the cg13471560 CpG site within the *CRNDE* gene. PCR product length: 250 bp; PCR conditions: 95 °C – 5 min, (94 °C – 45 s, 72 °C – 30 s, 72 °C – 45 s) x 40, 72 °C – 7 min, 4 °C – ∞; sequencing conditions: 95 °C – 2 min, (96 °C – 10 s, 70 °C – 5 s, 70 °C – 4 min) x 35, 4 °C – ∞.
CRNDEmR4	CATACACACTTCTTACATACACACTAATAA	
